# The Application of Resolvin D1-Loaded Gelatin Methacrylate in a Rat Periodontitis Model

**DOI:** 10.3390/pharmaceutics17010016

**Published:** 2024-12-25

**Authors:** Zhe Xing, Jing Liu, Jiazheng Cai, Xiaofeng Jiang, Jingwen Liang, Masahito Fujio, Elin Hadler-Olsen, Jing Wang, Alpdogan Kantarci, Ying Xue

**Affiliations:** 1 School of Stomatology, Lanzhou University, Lanzhou 730000, China; 2 Key Laboratory of Dental Maxillofacial Reconstruction and Biological Intelligence Manufacturing, Lanzhou University, Lanzhou 730000, China; 3 Clinical Research Center for Oral Diseases, Lanzhou University, Lanzhou 730000, China; 4 Department of Medical Biology, Faculty of Health Sciences, UiT the Artic University of Norway, 9037 Tromsø, Norway; 5 Department of Stomatology Center, Suining Central Hospital, Suining 629099, China; 6 Department of Oral and Maxillofacial Surgery, Graduate School of Medicine, Nagoya University, 65 Tsurumai-cho, Showa-ku, Nagoya 466-8550, Japan; 7 The Public Dental Health Service Competence Center of Northern Norway, 9271 Tromsø, Norway; 8 The Forsyth Institute, Cambridge, MA 02142, USA; 9 School of Dental Medicine, Harvard University, Boston, MA 02115, USA; 10 Department of Clinical Dentistry, Faculty of Health Sciences, UiT the Artic University of Norway, 9037 Tromsø, Norway

**Keywords:** periodontitis, RvD1 complexed with GelMA, drug release, inflammation, tissue regeneration

## Abstract

**Objective:** To evaluate the drug release, cytocompatibility with periodontal ligament cells (PDLCs), and therapeutic efficacy of GelMA hydrogel loaded with resolvin D1 (RvD1) in treating rat periodontal inflammation and alveolar bone damage. **Methods:** An RvD1 complexed with GelMA was prepared, and its release kinetics and compatibility with PDLCs were assessed. Rats with induced periodontitis were treated weekly with topical applications of vehicle, GelMA, RvD1, or RvD1 complexed with GelMA for four weeks. Periodontal inflammation and tissue regeneration were evaluated using quantitative PCR (qPCR) and histochemical staining, while alveolar bone repair and regeneration were analyzed through micro-CT. **Results:** The RvD1 complexed with GelMA effectively released RvD1 and enhanced the proliferation and viability of PDLCs. Compared to RvD1 alone, treatment with RvD1 complexed with GelMA significantly reduced inflammatory cell infiltration, TNF-α and RANKL expression, and osteoclast formation in periodontal tissues. Additionally, it promoted the expression of specific anti-inflammatory and regenerative markers. Micro-CT analysis confirmed significant new bone formation in the RvD1 complexed with GelMA-treated group. **Conclusions:** RvD1 complexed with GelMA provides sustained drug release and biocompatibility, effectively resolves periodontal inflammation, and promotes tissue regeneration in periodontitis.

## 1. Introduction

Periodontitis is a prevalent oral disorder influenced by both genetic and environmental factors. The interaction between plaque bacteria and the biologically active mediators of the host’s immune response gradually leads to the progressive destruction of the supporting tissues of teeth [1,2]. Recent studies have identified a correlation between periodontitis and systemic diseases, such as rheumatoid arthritis [3], diabetes [4], and pregnancy [5], all of which are common chronic inflammatory conditions. A dysregulated inflammatory response is the primary cause of periodontitis. The resolution of inflammation is a highly coordinated proactive process regulated by specialized pro-resolving lipid mediators (SPMs) [6]. SPMs, metabolites produced by specific lipoxygenases, facilitate the resolution of inflammation and restore biological function, avoiding the adverse events or resistance often associated with NSAIDs [7].

D-series resolvins (RvDs) are derived from docosahexaenoic acid (DHA), produced during the receding phase of inflammation. They may attenuate pro-inflammatory mediator responses, limit neutrophil infiltration, exhibit excellent anti-inflammatory effects in vivo, and promote the resolution of inflammation and tissue repair [8,9]. Inflammatory response and bone remodeling are inextricably linked processes. Bone remodeling is mediated by the recruitment of immune cells and a variety of growth factors and cytokines that promote the expression of mesenchymal cells [10]. RvD1 signals through specific G protein-coupled receptors, down-regulating inflammatory cytokines by inhibiting the activation of NF-κB signaling. This results in anti-catabolic effects in diseases characterized by bone loss and tissue destruction, positioning RvD1 as a potential treatment option for chronic inflammatory diseases such as periodontitis [11,12]. However, the rapid metabolism of RvD1 necessitates prolonging its action in vivo to improve clinical effectiveness.

A variety of drugs, including antibiotics, have been used as adjunctive therapy to maintain the long-term effects of periodontitis in clinical and research studies. However, their extended use may lead to a range of adverse effects, such as antibiotic resistance and suboptimal therapeutic concentrations [13,14]. In recent years, the development of safe and effective topical drug delivery systems has been explored for the treatment of periodontitis to achieve favorable therapeutic outcomes. These systems offer benefits such as flexible use, lower doses and dosing frequency, and improved patient compliance [15,16]. Periodontal drug delivery systems are designed to improve periodontal tissue health by regulating the absorption and release of therapeutic agents directly at the site of action through various carriers [17]. These delivery carriers include chitosan [18], cellulose [19], gelatin proteins [20], polylactic acid [21], and their combinations. Limitations for these systems included low bioavailability and quick drug release [22]. Hydrogel shows excellent water absorption, modifiability, and controlled-release properties and has been widely used in biomedical applications such as tissue regeneration, drug delivery systems, and periodontal applications [23]. Gelatin methacrylate (GelMA) is produced by the reaction of gelatin with methacrylic anhydride (MA) due to its biocompatibility, photocrosslinking ability, and multifunctional properties. Thus, GelMA shows great potential for periodontal tissue regeneration [24]. A low concentration of GelMA has a relatively loose-meshed structure that can promote cellular interactions [25].

This study assesses the controlled-release capabilities and functional effects of RvD1 complexed with GelMA, particularly its role in resolving periodontal inflammation and enhancing tissue regeneration.

## 2. Materials and Methods

### 2.1. Synthesis of GelMA Hydrogel and RvD1 Complexed with GelMA

GelMA hydrogels (EFL, Suzhou, China) were configured according to the manufacturer’s instructions. In brief, a 0.25% (*w*/*v*) initiator standard solution was prepared by mixing 20 mL of phosphate-buffered saline (PBS) with 0.05 g of photoinitiator, which was then mixed with GelMA to a final concentration of 5% (*w*/*v*) GelMA. The solution was sterilized using a sterile 0.22 µm filter. For the RvD1 and GelMA group, GelMA was first cured with LED light (Woodpecker, Guilin, China) for 30 s, and then RvD1 was added at a concentration of 100 ng/mL. For the RvD1 complexed with the GelMA group, 100 ng/mL RvD1 was directly incorporated into the GelMA solution to form a slow-release complex. These complexes were subsequently stored at −20 °C and cured for 30 s before use.

### 2.2. Drug Release

For RvD1 release kinetics, 200 µL RvD1 complexed with GelMA was added to each well in 48-well plates (*n* = 4) and polymerized under LED light for 30 s. The plates were then incubated at 37 °C in a shaker and kept in the dark. PBS was replaced daily and at specified intervals (1, 3, 7, 12, 18, and 24 days), and 500 µL from each well was sampled and replenished with PBS. RvD1 levels in the supernatant were quantified using the RvD1 ELISA KIT (Jianglai, Shanghai, China).

### 2.3. Cell Culture and Cell Proliferation Assay

PDLCs were acquired and cultured as previously described [26], with ethical approval (LZUKQ-2020-026). The PDLCs were cultured in an α-modified minimal essential medium (α-MEM, Pittsburgh™, Pittsburgh, PA, USA) supplemented with 10% fetal bovine serum (FBS, Abwbio, Shanghai, China) and 1% Penicillin-Streptomycin. The cells were incubated in 5% CO_2_ in 100% humidity at 37 °C. For cell proliferation assays, 200 µL of GelMA, RvD1 and GelMA, or RvD1 complexed with GelMA were added to 48-well plates and light-cured for 30 s. PDLCs were seeded at 2.5 × 10^5^ cells/well on GelMA, RvD1 and GelMA, or RvD1 complexed with GelMA and cultured for 1, 3, or 7 days. Cell proliferation was assessed using the Cell Counting Kit-8 (CCK-8, AbMole BioScience, Houston, TX, USA). Absorbance was measured at 450 nm with an Infinite 200Pro microplate reader (Tecan, Grödig, Austria).

### 2.4. Experimental Periodontitis and Topical Application of RvD1 Complexed with GelMA

Male Sprague Dawley rats (180–200 g, 6–8 weeks) were used, with ethical approval (LZUKQ-2021-035). A control group (5 rats) received no treatment, while others had 4-0 silk threads (Baohe, Shanghai, China) ligated around maxillary first molars to induce periodontitis for 4 weeks. Post-ligation, rats were divided into five groups: periodontitis (P; 7 rats), periodontitis+PBS (vehicle; 4 rats), periodontitis+RvD1 (9 rats), periodontitis+GelMA (9 rats), and periodontitis+RvD1 complexed with GelMA (9 rats). The periodontitis group was sacrificed to assess lesions. The remaining groups received weekly treatments of PBS, RvD1, GelMA, or RvD1 complexed with GelMA for 20 µg, followed by UV light exposure for 30 s.

### 2.5. Histomorphometry

After four weeks, experimental and control group rats were euthanized, and periodontal and maxillary bone tissues were harvested. Left-side tissues were used for histomorphometry, while right-side tissues were preserved in RNA later. The specimens (*n* = 4) were stained with 1% methylene blue (Melan, Dalian, China) to assess bone resorption at six sites around the maxillary first molar. Paraffin sections were stained with hematoxylin and eosin staining (HE) to identify inflammatory cells of different morphologies. Inflammation was evaluated by counting the total number of inflammatory cells, including neutrophils, lymphocytes, eosinophils, and macrophages, within the lamina propria above the alveolar bone crest between the distal root of the maxillary first molar and the mesial root of the second molar. This was done using three randomly selected images per sample for each experimental group at 40× magnification. Osteoclast activity was assessed using tartrate-resistant acid phosphatase (TRAP), which has red cytoplasm and blue nuclei. The number of positive osteoclasts was counted in the alveolar bone between the maxillary first molar distal root to the second molar mesial root from three randomly selected images of each sample in each experimental group at 40× magnification. Masson staining was employed to detect new collagen deposition (blue) and new bone formation (red) after treatment.

### 2.6. Micro-Computed Tomography (Micro-CT) Analysis

Micro-CT was employed to analyze subtle changes in rat alveolar bone structure. Rat maxillary alveolar bones were scanned using a BRUKER Micro-CT SkyScan 1176 (Bruker, Billerica, MA, USA) at a resolution of 17.76 µm, X-ray voltage of 65 kV, and source current of 385 µA.

The analysis focused on the sagittal plane from the distal root of the first molar to the mesial root of the second molar. Measured micro-parameters included bone mineral density (BMD), relative bone volume (BV/TV), trabecular thickness (Tb.Th), separation rate (Tb.Sp), and number (Tb.N), revealing detailed bone microarchitecture and treatment responses.

### 2.7. Quantitative Polymerase Chain Reaction (q-PCR) Analysis

q-PCR analysis of inflammation and osteogenesis genes was performed as demonstrated in our previous article [27]. Primer details are provided in Table 1. The q-PCR utilized glyceraldehyde 3-phosphate dehydrogenase (GAPDH) as the internal reference. Gene expression levels were calculated using the 2^−ΔΔct^ method and analyzed with GraphPad 9 software, with each experiment conducted in triplicate.

### 2.8. Statistical Analysis

All experiments were repeated at least three times; all measurements were expressed as mean ± standard deviation and statistically analyzed with GraphPad Prism software. A *t*-test or ANOVA analysis was performed for statistical evaluation, and differences were considered statistically significant at *p* < 0.05.

## 3. Results

### 3.1. Release Kinetics Study of RvD1

**Figure 1:**
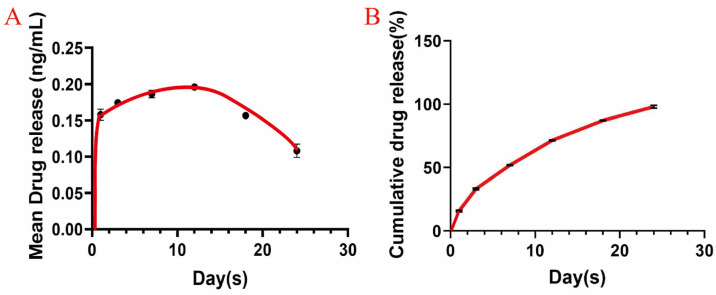
The release of RvD1 complexed with GelMA over time in vitro. (**A**) Mean drug release from RvD1 (ng/mL); (**B**) cumulative release of RvD1 (%).

The mean release of RvD1, as detected by ELISA, is illustrated in Figure 1A. Figure 1B shows the cumulative release over time. RvD1 release rapidly increased on the first day and subsequently stabilized. The release of RvD1 complexed with GelMA had a good release profile for two weeks, suggesting that RvD1 complexed with GelMA was suitable for a controlled release of a stable and localized concentration of the delivery of a bioactive compound.

### 3.2. RvD1 Complexed with GelMA Promotes Cell Proliferation

**Figure 2:**
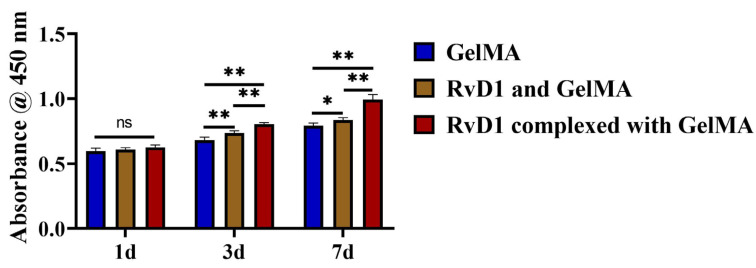
CCK-8 experiments showed the proliferation of PDLCs in the GelMA, RvD1 and GelMA, and RvD1 complexed with GelMA groups. ** *p* < 0.01; * *p* < 0.05; ns: no significant difference.

CCK-8 results are shown in Figure 2; cell proliferation in the RvD1 complexed with GelMA group was higher compared to both the GelMA group and the RvD1 and GelMA group. The RvD1 complexed with GelMA group showed significantly higher proliferation than the remaining two groups on day 7 (*p* < 0.05). These results indicate that RvD1 complexed with GelMA can promote PDLC proliferation.

### 3.3. RvD1 Restores Alveolar Bone Loss in Experimental Periodontitis by Regulating Osteogenesis-Related Factor Expression

**Figure 3:**
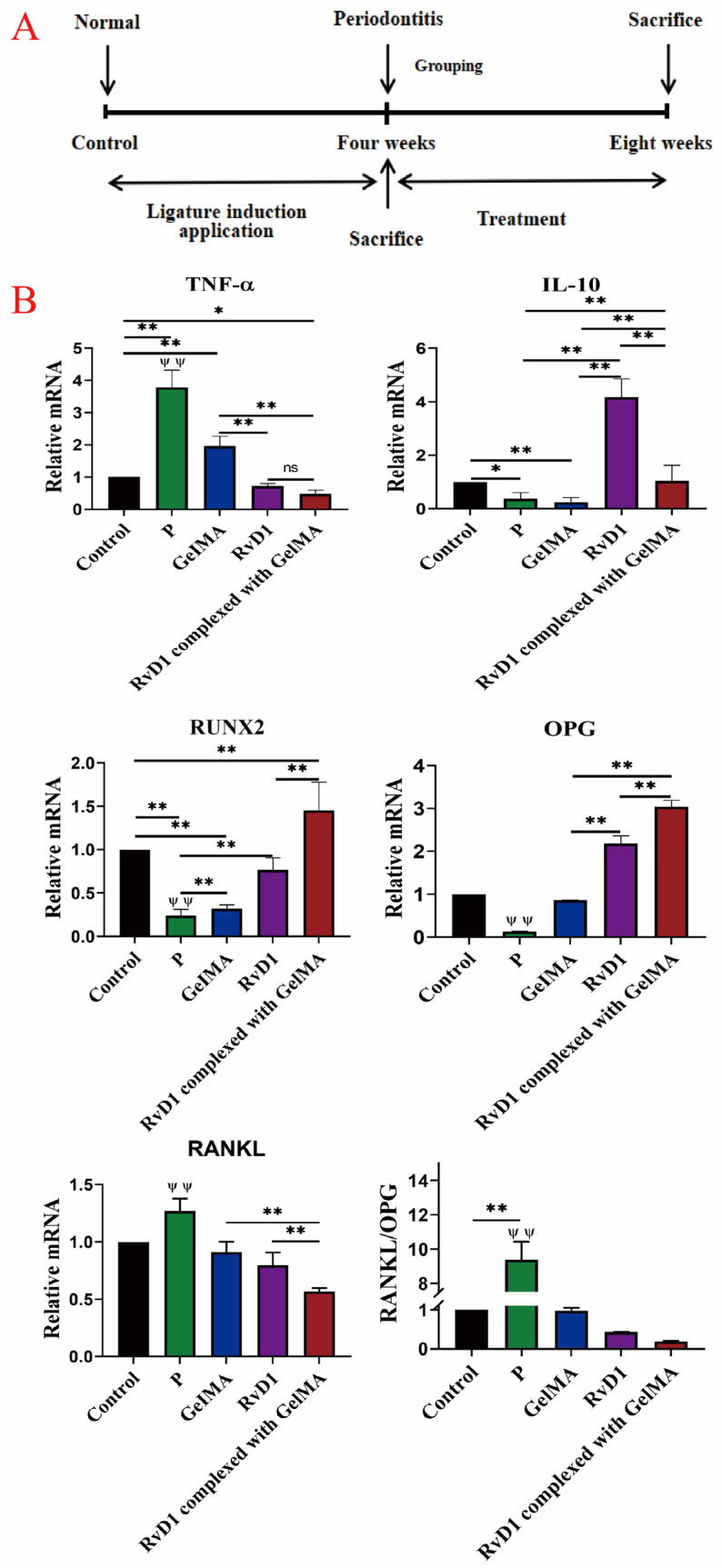
RvD1 and its complex alters the expression of inflammatory factors. (**A**) Timeline of animal experiments. (**B**) q-PCR to detect the relative mRNA expression of OPG, RUNX2, RANKL, IL-10, and TNF-α in the periodontal tissue of the maxillary first molars in each group after 4 weeks of administration. ψψ means P group vs. GelMA group, RvD1 group, and RvD1 complexed with GelMA group; **, ψψ *p* < 0.01; * *p* < 0.05; ns: no significant difference.

Figure 3A shows the experimental procedure in which PBS, RvD1, GelMA, and RvD1 complexed with GelMA were injected weekly into the periodontal pockets of maxillary first molars after the rat periodontitis model was established. q-PCR results (Figure 3B) indicate that the expression of the pro-inflammatory cytokine TNF-α was down-regulated, while the anti-inflammatory cytokine IL-10 was up-regulated in the RvD1 and RvD1 complexed with GelMA groups, suggesting resolution of the periodontal inflammatory response with RvD1 treatment. Additionally, mRNA expression of osteogenesis-related factors showed that RANKL expression was significantly reduced (*p* < 0.01), and the expression of OPG and RUNX2 was increased (*p <* 0.01), particularly in the RvD1 complexed with GelMA group, highlighting its effectiveness in resolving periodontal inflammation.

**Figure 4:**
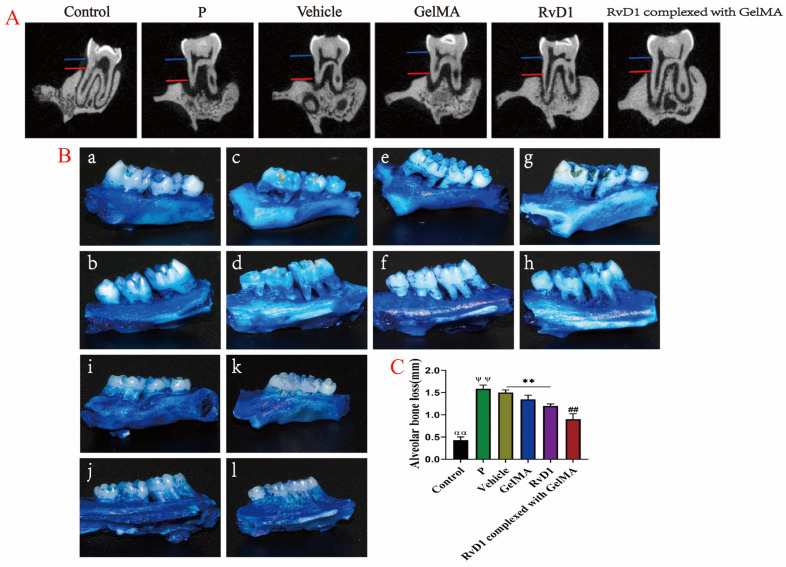
RvD1 and its complex restore alveolar bone loss. (**A**) Representative micro-CT images of coronal surfaces of maxillary first molars showing the effect on attachment loss after treatment. (**B**) Methylene blue staining of rat maxillary bone samples: (**a**,**b**) for Control group; (**c**,**d**) for P group; (**e**,**f**) for Vehicle group; (**g**,**h**) for GelMA group; (**i**,**j**) for RvD1 group; (**k**,**l**) for RvD1 complexed with GelMA group; (**a**,**c**,**e**,**g**,**i**,**k**) for the buccal side and (**b**,**d**,**f**,**h**,**j**,**l**) for the lingual side of teeth. (**C**) Quantitative analysis of alveolar bone loss in all samples. αα means: Control group vs. P group, Vehicle group, GelMA group, RvD1 group, and RvD1 complexed with GelMA group; ## means: RvD1 complexed with GelMA group vs. GelMA group, Vehicle group, and RvD1 group; ψψ means: P group vs. GelMA group, RvD1 group, and RvD1 complexed with GelMA group; **, αα, ##, ψψ *p* < 0.01.

Treatment with RvD1 or RvD1 complex restored periodontal attachment, as demonstrated in Figure 4A. Digital images of the maxillary alveolar bone stained with methylene blue (Figure 4B) showed that significant alveolar bone loss occurred on the buccal and palatal sides after periodontitis induction. Post-treatment, new alveolar bone formation was observed, with RvD1 complexed with GelMA showing the most significant enhancement in bone formation (*p* < 0.01). These findings suggest that the RvD1 complexed with GelMA treatment not only effectively controls periodontal inflammation but also facilitates the restoration of alveolar bone loss more effectively than RvD1 alone.

### 3.4. RvD1 and Its Complexes Promote Alveolar Bone Repair in Rats

**Figure 5:**
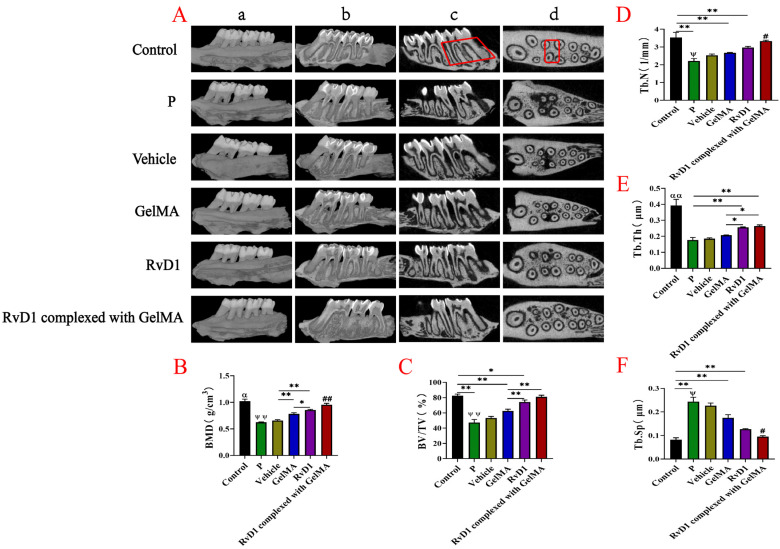
RvD1 and its complex protect the alveolar bone of rats from periodontitis. (**A**) Micro-CT for 3D reconstruction of rat maxillary alveolar bone (**a**,**b**) and CT scans (sagittal view (**c**) and cross-sectional view (**d**)); (**B**) bone mineral density; (**C**) relative bone volume; (**D**) trabecular number; (**E**) trabecular thickness; (**F**) trabecular separation. α/αα means: Control group vs. P group, Vehicle group, GelMA group, RvD1 group, and RvD1 complexed with GelMA group; #/## means: RvD1-GelMA group vs. GelMA group, Vehicle group, and RvD1 group; ψ/ψψ means: P group vs. GelMA group, RvD1 group, and RvD1 complexed with GelMA group; **, αα, ##, ψψ *p* < 0.01; *, α, #, ψ *p* < 0.05.

Figure 5A illustrates the analysis of the internal microstructure of each group conducted by micro-CT. Figure 5A(a,b) show the three-dimensional reconstructed images of the maxillary bone, which can visualize the changes in samples of alveolar bone. Significant alveolar bone resorption was seen in rats after 4 weeks of ligation. After 4 weeks of treatment, the RvD1 and RvD1 complexed with GelMA could reverse bone destruction. Micro-CT was also used to quantify the alveolar bone micro-parameters (BMD, BV/TV, Tb.Th, Tb.Sp, and Tb.N) for each group in a specific area (the red quadrilateral area in Figure 5A(c,d)). In Figure 5B–F, the BMD, BV/TV, Tb.Th, and Tb.N of the first molar were reduced (*p* < 0.05), and Tb.Sp was increased (*p* < 0.05) after ligation. Compared with the periodontitis group, the BMD, BV/TV, Tb.Th, and Tb.N increased, and Tb.Sp decreased in RvD1 and RvD1 complexed with GelMA groups after treatment, collectively suggesting that RvD1 and its complex can repair bone loss with new high-density bone.

### 3.5. RvD1 and Its Complex Have a Restorative Effect on Periodontal Damage Caused by Experimental Periodontitis in Rats

**Figure 6:**
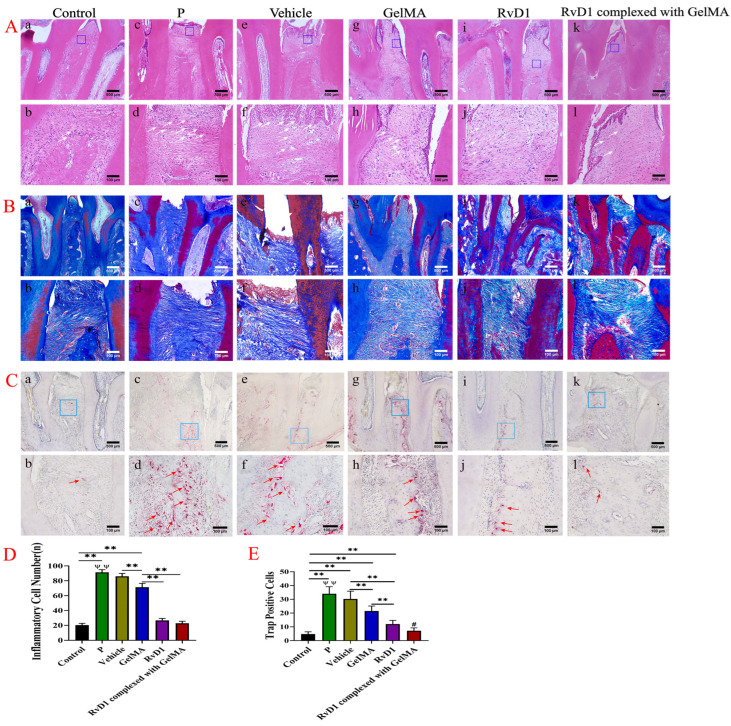
Inflammation in periodontal tissues of rats was significantly alleviated and promoted periodontal tissue regeneration after RvD1 and its complex treatment. (**A**) Maxillary bone samples were collected for HE staining, and many inflammatory cells (white arrows) were visible at the ligation site in the P group; periodontitis was significantly alleviated after 4 weeks of treatment with RvD1 and its complex. The small boxes represent the locations where images were captured at 40× magnification. (**B**) Masson section staining was used to evaluate the regeneration of periodontal fibrous connective tissue and alveolar bone promoted by the action of RvD1 and RvD1 complexed with GelMA. (**C**) Evaluation of osteoclast (red arrow) expression in alveolar bone by trap section staining. (**D**) Statistical analysis of the number of inflammatory cells in periodontal tissues for each group (number of inflammatory cells within the blue quadrilateral area of the figure). (**E**) The results of quantitative analysis of osteoclasts (the number of positive cells in the blue quadrant of the figure). (**a**,**b**) for Control group; (**c**,**d**) for P group; (**e**,**f**) for Vehicle group; (**g**,**h**) for GelMA group; (**i**,**j**) for RvD1 group; (**k**,**l**) for RvD1 complexed with GelMA group; (**a**,**c**,**e**,**g**,**i**,**k**) for magnification 40×, scale bar 500 µm; (**b**,**d**,**f**,**h**,**j**,**l**) for magnification 200×, scale bar 100 µm. # means: RvD1 complexed with GelMA group vs. GelMA group, Vehicle group, and RvD1 group; ψψ means: P group vs. GelMA group, RvD1 group, and RvD1 complexed with GelMA group; **, ψψ *p* < 0.01; # *p* <0.05.

Figure 6A shows HE-stained sections of periodontal tissues in rats. Figure 6A shows considerable inflammatory cell infiltration, degradation of fibrous connective tissue, and alveolar bone resorption in the periodontal tissue following ligation. Figure 6D shows the number of inflammatory cells in the blue quadrilateral area after therapy. The vehicle and GelMA treatments had minimal therapeutic effects on periodontitis, with persistent severe inflammatory cell infiltration in the vehicle group (*p* > 0.05) and slight alleviation in the GelMA group (*p* < 0.05). However, the condition significantly improved in RvD1 and RvD1 complexed with GelMA groups, with decreasing inflammatory cell number (*p* < 0.05), partial restoration of the irregular bone surface, and new bone forming at the bone defect.

The fibrous connective tissue that connects teeth and alveolar bone is damaged during periodontitis; we used Masson staining to assess the alterations (Figure 6B). The connective tissue layer was destroyed severely, with an irregular fiber organization in the periodontitis group (Figure 6B(c,d)). Treatment with vehicle did not improve periodontal inflammation and tissue regeneration (Figure 6B(e,f)); GelMA reduced inflammation, and some disorganized and scattered new collagen fiber creation was visible, but there was no significant new bone formation (Figure 6B(g,h)). For alveolar bone production, densely structured and well-ordered new collagen fibers were seen in sections after treatment with RvD1 (Figure 6B(i,j)) and RvD1 complexed with GelMA (Figure 6B (k,l)).

Trap staining was used to identify the osteoclasts in periodontitis (Figure 6C). In Figure 6C(c,d), the periodontitis group had clear alveolar bone loss and osteoclasts in bone resorption traps. There were also osteoclasts in the vehicle group, a sign of ongoing inflammation (Figure 6C(e,f)). GelMA reduced bone resorption and tended to encourage osteogenesis. Osteoclasts were rarely observed following treatment with RvD1 and RvD1 complexed with GelMA (Figure 6C(i–l), respectively). Quantitative analysis of osteoclast counts in the blue quadrilateral area (Figure 6E) revealed drastically reduced osteoclast counts treated with RvD1 complexed with GelMA (*n* = 7.667 ± 2.121) and RvD1 (*n* = 11 ± 2.828), especially in RvD1 complexed with GelMA (*p* < 0.05).

## 4. Discussion

Resolvin, which are endogenous lipid mediators, activate inflammatory resolution pathways and promote tissue recovery, indicating that controlling inflammation effectively promotes wound healing and tissue restoration [28]. In vivo, specific members of the resolvin family control the degree of inflammation and the time to restore tissue homeostasis [29]. In vitro, RvD1 significantly promotes PDLC proliferation and periodontal tissue recovery [30], acting at injury sites in a dose- and time-dependent manner [31]. This study investigated the integration of 100 ng/mL RvD1 in GelMA hydrogels, examining its impact on inflammatory resolution and tissue regeneration in rat periodontitis. RvD1 complexed with GelMA, a topical controlled-release system, was found to significantly enhance the resolution of experimental periodontitis and promote tissue regeneration.

To confirm the sustained-release effect, the release capability of RvD1 complexed with GelMA was assessed in vitro, showing stable RvD1 release over two weeks and suggesting reliability as a controlled-release system. In vivo, variables such as pH [32], temperature [33], enzymes [34], and local activity in the oral environment can accelerate drug release. Based on the results of RvD1 release kinetic studies, the topical application of the drug once a week in the periodontium can maintain a more stable drug concentration and give a more accurate drug delivery time in this experiment. Biocompatibility is crucial when applying hydrogels in drug delivery systems, so the CCK-8 assay was used to evaluate the potential cytocompatibility of RvD1 complexed with GelMA on PDLCs. After the PDLCs were inoculated onto various hydrogel surfaces, the cell counts increased during 7 days in all groups, and the RvD1 complexed with GelMA group showed the greatest increase. It is suggested that the controlled-release complex formed by RvD1 and GelMA can be more effective in promoting PDLC proliferation and has good cytocompatibility for the growth of PDLCs.

Optimizing the dose for the desired effect is challenging within a single animal experiment, representing a potential limitation of our study, as we were only able to compare a low-dose concentration. Despite this, we observed promising effects on wound healing and osteogenesis at this low concentration. Of course, determining the optimal dose requires alignment with the wound healing curve in humans. Ideally, this curve shows low pro-resolving concentrations during the early inflammatory phase and higher levels during the mid-soft callus phase [35]. Our future research will focus on controlling drug concentrations at different stages of tissue healing to better align with these requirements.

Biomaterials trigger a series of material-related host responses after implantation at the action site, which regulates the local immune microenvironment and impacts how damaged tissues repair around the implant. Specific chemical components in biomaterials can produce a toxic response at the implantation site, thereby altering the early stages of tissue healing and subsequent reaction processes at the injury site [36,37]. To mitigate any potential cytotoxic effects of both GelMA and RvD1, we carefully optimized the concentrations to ensure they are within a range that is effective yet non-toxic to cells. In future studies, we plan to further assess the long-term biocompatibility and safety of the materials in a physiological environment.

RvD1 can attenuate pro-inflammatory mediator responses, limit excessive neutrophil infiltration, and enhance macrophage action; this will promote inflammation resolution and repair damaged tissues [38,39]. The tail vein injection of RvD1 protected lung injury in mice by reducing TNF-α, up-regulating M2-type macrophages, and alleviating acute lung injury [40]. The inflammatory cytokines TNF-α and IL-10, secreted by macrophages, are crucial for maintaining the dynamic balance between pro-inflammation and regression [41]. The healing of the periodontium, particularly in the context of periodontitis, involves complex interactions between various factors. The healing of mucosal tissues, including ulcerations, is influenced by inflammatory cytokines, endogenous growth hormone, and insulin-like growth factor-1 [42]. Bone healing requires not only the resolution of inflammation but also the stimulation of osteogenic factors to promote regeneration. In this study, during RvD1 complexed with GelMA treatment, a significant reduction in inflammatory cell infiltration and inflammatory cell count was observed in periodontal tissues, leading to lower expression of TNF-α and an increase in IL-10. The inflammation resolution was more pronounced in the RvD1 complexed with GelMA group than in RvD1, presumably because the complex could sustain stable concentrations of RvD1 and more efficiently activate anti-inflammatory indexes, implying that the periodontal inflammatory response may have ended earlier when RvD1 was incorporated into the controlled-release delivery system. These findings suggest that the early elimination of periodontal inflammation promotes periodontal tissue repair.

In the inflammatory response phase, macrophages release cytokines, stimulating osteoclasts, inhibiting osteoblast function, and causing alveolar bone loss [43]. RvD1 could also reduce the expression of TNF-α, RANKL, and TRAP and reduce bone destruction in mice with joint inflammation [44]. The histomorphometry results revealed that the alveolar bone level in the RvD1 and RvD1 complexed with GelMA groups was improved, the number of osteoclasts decreased, OPG and RUNX2 expression levels increased, and RANKL declined in periodontal tissues. The RvD1 complexed with GelMA group changes were more obvious than for RvD1, and the RANKL/OPG ratio was altered. According to the micro-CT results, RvD1 complexed with GelMA positively impacted the alveolar bone micro-parameters, including bone surface area, bone density, and bone intensity. Our findings indicate that RvD1 complexed with GelMA could restore alveolar bone loss more effectively than RvD1 alone; this could be due to the inhibition of the NF-κB signaling pathway, which regulates the RANKL-OPG axis.

Macrophages play an important role in this process [45]. Therefore, the local characterization of different biomaterials and their effects on the functional patterns of macrophages play an important role in the development and study of the specific modes of action of different materials in the process of organismal immunomodulation [46,47]. RvD1-loaded bionic anti-inflammatory nano-capsules could regulate the inflammatory response, enhance the polarization of M2 macrophages, and encourage bone growth in the bone defect area [48]. In our study, the highest levels of IL-10 observed in the RvD1 group may be attributed to the sudden introduction of RvD1, which could have triggered an enhanced anti-inflammatory response. OPG and RUNX2 expression increased after GelMA action, while IL-10 decreased considerably compared to the periodontitis group; this expression was more pronounced in the RvD1 complexed with GelMA group. We consider that the RvD1 complex with GelMA likely provides a sustained release of RvD1, which may create a more prolonged and stable environment conducive to osteogenic activity. This sustained release could enhance the expression of RUNX2 and OPG over time, promoting bone regeneration and remodeling. Moreover, we hypothesize that RvD1 complexed with GelMA may promote periodontal inflammation regression and tissue restoration by altering the functional phenotype of macrophages. Our ongoing studies have explored the effects of RvD1 on macrophages using RNA sequencing and cellular experiments.

In this study, GelMA hydrogel was utilized as the controlled-release carrier of RvD1. It was found that the RvD1 complexed with GelMA exhibited an effective controlled-release profile. Animal experiments further confirmed that it could reduce periodontal inflammation and promote the recovery of periodontal tissues in rats. In future, we plan to investigate the specific mechanisms of this delivery system in vitro, assess its feasibility for clinical application, and explore its role in improving periodontal inflammation and promoting tissue regeneration. Additionally, further in vivo and in vitro experiments are necessary to elucidate the specific molecular mechanisms by which RvD1 complexed with GelMA influences osteoblasts and osteoclasts.

## 5. Conclusions

This study showed that RvD1 complexed with GelMA has a sustained-release effect and biocompatibility. The controlled-release complex outperformed RvD1 alone in reducing inflammation and promoting periodontal tissue repair when given topically to a rat experimental periodontitis model. Our findings support the idea that the topical administration of RvD1 in a controlled-release GelMA hydrogel is a promising drug delivery system for the efficient treatment of periodontitis.

## Figures and Tables

**Table 1:** Sequences of forward and reverse primers of selected genes designed for PCR.

Gene	Forward Primer, 5′-3′	Reverse Primer, 5′–3′
GADPH	GGCACAGTCAAGGCTGAGAATG	ATGGTGGTGAAGAVGVCAGTA
TNF-α	GGCGTGTTCATCCGTTCTC’	CTTCAGCGTCTCGTGTGTTTCT
IL-10	CAGACCCACATGCTCCGAGA	CAAGGCTTGGCAACCCAAGTA
RUNX2	CATGGCCGGGAATGATGAG	TGTGAAGACCGTTATGGTCAAAGTG
RANKL	GCAGCATCGCTCTGTTCCTGTA	GCATGAGTCAGGTAGTGCTTCTGTG
OPG	CTCATCAGTTGGTGGGAATGAAGA	ACCTGGCAGCTTTGCACAATTA

## Data Availability

The original contributions presented in this study are included in the article. Further inquiries can be directed to the corresponding authors.
